# Hygiene perception changes during the influenza A H1N1 pandemic in Germany: incorporating the results of two cross-sectional telephone surveys 2008–2009

**DOI:** 10.1186/1471-2458-13-959

**Published:** 2013-10-16

**Authors:** Gerald Meilicke, Klaus Riedmann, Walter Biederbick, Ute Müller, Traugott Wierer, Cornelius Bartels

**Affiliations:** 1 Robert Koch Institute, Berlin, Germany; 2 Federal Ministry of Health, Berlin, Germany; 3 Federal Office of Civil Protection and Disaster Assistance, Bonn, Germany; 4 Forsa Gesellschaft für Sozialforschung und statistische Analysen mbH, Berlin, Germany; 5 Statistik-Service Dr. Gladitz, Berlin, Germany; 6 European Centre for Disease Prevention and Control, Stockholm, Sweden

## Abstract

**Background:**

The federal campaign *Wir gegen Viren* [Us against viruses] promoted hygiene in Germany during the influenza A H1N1 pandemic in 2009. The intervention aimed to encourage people to protect themselves against respiratory infections by simple means of hygiene behaviour. Quantitative research was carried out to outline changes in hygiene perception of the population over time, and to find out whether the potential hygiene perception changes were consistent to the federal campaign about hygiene or not.

**Methods:**

To determine changes in the hygiene perception of the population, two cross-sectional telephone surveys were held, each one with n = 2006 participants. The initial survey was carried out before the influenza A H1N1 pandemic in calendar week 49–51 in 2008 and the second in week 48 in 2009 directly after the peak of the pandemic in Germany. The questionnaire contained indicators about perceived hand hygiene efficacy, preference for coughing into the sleeve, propensity for presenteeism while showing symptoms of a cold and acceptance of hygiene masks.

**Results:**

The proportion of people who perceive the efficacy of hand washing as “very good” increased significantly from 50.9% in 2008 to 61.1% in 2009. The proportion of people who perceive coughing into the sleeve as the best way to cough increased even more dramatically from 4.8% in 2008 to 38.3% in 2009. In contrast the propensity for presenteeism decreased significantly: The proportion of people who state that they always report to work while they show symptoms of a cold decreased from 50.8% in 2008 to 40.9% in 2009. Acceptance of hygiene masks has not changed significantly from 2008 to 2009.

**Conclusions:**

The results revealed changes in hygiene perception during influenza A H1N1 pandemic in Germany. The changes we found are in accordance with the hygiene recommendations given by the federal campaign *Wir gegen Viren* [Us against viruses]. Results can constitute a practical benchmark for future research about hygiene perception and hygiene promotion for adults. A pivotal question is: does the increase in hygiene perception persist after the pandemic has ceased?

## Background

Germany, like other countries throughout the world, faced extraordinary challenges during 2009 regarding communication about hygiene. The emergence of the influenza A H1N1 viral strain created a higher demand for information, forcing public health professionals to disseminate consistent guidelines in a continuously changing pandemic situation [1-5]. Influenza A H1N1 started to become a public issue in Germany in calendar week 18. Until week 31, imported infections of influenza had a major role in influenza activity. From week 31 until week 42 the number of domestic infections increased strongly. In week 42 the main pandemic wave in Germany began, and reached a peak in week 47. By the end of 2009 the number of reported infections had strongly decreased again [6].

Hygiene promotion is considered a crucial public health activity not only in pandemic situations but also in perennial infection control [7-9]. In week 14, immediately before the influenza A H1N1 pandemic became a public health issue, federal public health institutions in Germany introduced the hygiene campaign *Wir gegen Viren* [Us against viruses] [10]. The recommendations focused on non-pharmaceutical interventions to protect people from seasonal respiratory infections and pandemic influenza. The recommendations embraced the main hygiene issues, firstly that hand washing can reduce the risk of infection, secondly that it is recommended to cough into your sleeve, thirdly that you are supposed not to go to work when you caught a cold and fourthly that there is yet a lack of evidence for the efficacy of hygiene masks during an influenza pandemic. The information was featured by a video spot, a poster, a flyer, a website and stickers [10].

This article reports the results of two cross-sectional telephone surveys carried out to get a better understanding of the perception of the aforementioned four hygiene recommendations in the population of Germany. The aim of the surveys was to discover, whether or not there were changes in perceptions of the hygiene issues with time and to find out whether the perception was consistent to institutional recommendations about hygiene or not.

## Method

### Participants

Subjects of the surveys were inhabitants of Germany aged 18 or above, capable of speaking German and living in a private household with a telephone connection.

### Polling company

Sampling and telephone interviews were provided by the professional German polling company *forsa Gesellschaft für Sozialforschung und Statistische Analysen mbH* as part of their continuous omnibus survey [11].

### Sampling

The respondents were selected by a multi-level probability sample. The core of the sample is an artificially generated set of digit sequences. The set contains all the phone numbers in Germany, including both registered and non-registered numbers. Sampling was done randomly and all numbers had the same statistical probability of being included in the sample [12,13]. If the phone was unanswered, the number was tried again for a maximum of 10 times. When answered, the interviewers selected the respondent in the household by the birthday method. The interviewer asked the person who picked up the phone, which household member’s birthday was most recent. If this person could not be interviewed immediately, an appointment time was made. It was not permitted to substitute the selected person with another household member. If the selected person could not be interviewed at all, the household was no longer considered for the sample.

### Questionnaire

Corresponding with the four main recommendations during influenza A H1N1 pandemic we used four questions in our survey. For the first we asked respondents to tell us “How well can hand-washing reduce the risk of catching a cold?” Possible responses were “very good”, “good”, “somewhat”, “not at all” and “do not know”. For the second we asked “People cough in many different ways. Some cover their mouth with their hand, some cough into the sleeve of their coat or jacket or into a tissue, and others cough openly. In your opinion, which is the best way to cough?” Possible responses were “into the sleeve”, “into a tissue”, “into the hand”, “openly”, “none of the above” and “do not know”. For the third we asked “After you have caught a common cold, and start coughing and sneezing, would you still go to work?” Possible responses were “always”, “mostly”, “seldom”, “never”, “sometimes” and “do not know”. The answer option “sometimes” was never proposed by the interviewer and only noted if the participant mentioned it spontaneously. For the fourth we asked “In Japan, many people wear a hygiene mask when they have caught a cold to protect others from being infected. Would you favour more people in Germany doing so?” Possible responses were “I would favour a mask”, “I would not favour a mask” and “do not know”.

Before asking questions about hygiene, the respondents were asked about demographics including sex, age group and education. Age was classified into four groups: 18–29, 30–44, 45–59 and 60+ years old. Concerning education, we analysed groups with a 9-year education, 10-year education and 12 or more years of education.

Other questions in the field of pandemic influenza have been asked afterwards in the study but have been excluded from this article for brevity as well as clear focus on the main hygiene recommendations given to the population by the federal agencies during the influenza A H1N1 pandemic. Questions from other clients of the polling company, which are not reported, can have been asked due to the continuous omnibus approach. We arranged that no other questions in the same or a related field of hygiene and infection control of another of their clients were asked in the same interviews. Also more questions were asked about demographics, most notably the region of residence, as this besides sex, age group and education was to be used for weighting the results according to the population in Germany.

### Field

The initial telephone survey was held before the influenza A H1N1 pandemic in week 49–51 of 2008 with n = 2006, the number of respondents offered by the polling company. The second survey was carried out directly after the peak of the pandemic in Germany in week 48 of 2009, again with n = 2006. Response rates cannot be given exactly, due to the continuous sample process of the omnibus design. To provide a reference response rate for the omnibus sample of one whole year, the polling company evaluates a randomized sample of 10000 numbers. The sample is drawn on the first work day of each quarter of the year. The results of the four quarters together produce the annual average response rate reported as the percentage of all eligible people who answered the phone. This average response rate amounted 44% in 2008 and 45% in 2009.

Question 3 was only asked to working people (participants in question 3: 2008: n = 958; 2009: n = 979). Retired and unemployed people were not asked question 3.

To adjust differences in the composition of the sample and the distribution throughout the population, for each respondent a specific weight is calculated to match in terms of age, sex, region and education according to the population statistics of the Federal Statistical Office of Germany [11,14,15]. The determined weight factors are accounted into the further statistical analysis of the sample.

### Statistical analysis

The statistical significance of hygiene perception differences between 2008 and 2009 as well as demographic variables sex, age and education were analysed in a multivariate model of logistic regression.

As the inclusion of interaction effects between time and demographics into the model showed no significant interaction effects, we focus in this report on the main effects with odds ratios and 95% confidence intervals. SPSS 20 was used to analyse the data.

### Ethics statement

The forsa omnibus surveys applied guidelines and code of conduct of the ADM Arbeitskreis Deutscher Markt- und Sozialforschungsinstitute e.V. [Association of German market and social research institutes] [16]. Participants were informed about the general purpose and the voluntary nature of the surveys before verbal consent was obtained. Robert Koch Institute’s publication review and dual use potential review of the manuscript revealed no objections to this publication of study results.

## Results

### Hand washing

The perceived efficacy of hand washing increased from 50.9% in 2008 to 61.5% in 2009. Significantly more participants considered the infection control effect of hand-washing “very good”. Women estimated the infection control effect more frequently as “very good” than men. Older age groups estimated the effect higher than younger ones. Educational background showed no significant influence on how efficient hand washing was perceived.

### Coughing into the sleeve

The opinion about coughing into the sleeve changed dramatically during the period of study. In 2008 only 4.8% of the respondents considered it the best way to cough while in 2009 already 38.3% did so. Women prefer coughing into the sleeve slightly more frequent than men. Younger age groups more frequently thought that it was best to cough into the sleeve, compared with the oldest age group. There is also significant difference between the highest and the lowest education level, with the lowest education level being less likely to vote for coughing into the sleeve as best way to cough (Table 1).

**Table 1:** Multivariate analysis of factors associated with perceived efficacy of hand-washing and estimating into the sleeve as best way to cough (n = 4.012, pooled data week 49-51/2008 and week 48/2009)

	**Efficacy of hand-washing is very good**			**Into the sleeve is best way to cough**		
	**No./%**	**Odds ratio**	**Confidence interval 95%**	**No./%**	**Odds ratio**	**Confidence interval 95%**
**Year**						
2009	1230 (61.5)	*1.54	(1.31-1.80)	825 (38.3)	*13.07	(10.00-17.08)
2008	987 (50.9)	1		100 (4.8)	1	
**Sex**						
Female	1315 (61.1)	*1.47	(1.25-1.72)	549 (23.9)	*1.50	(1.21-1.85)
Male	902 (51.0)	1		376 (19.2)	1	
**Age**						
18-29	308 (50.4)	**0.66	(0.51-0.85)	171 (27.1)	*2.19	(1.54-3.12)
30-44	624 (54.2)	**0.77	(0.62-0.96)	337 (27.4)	*2.40	(1.80-3.20)
45-59	622 (55.7)	**0.79	(0.64-0.99)	229 (19.6)	**1.39	(1.04-1.87)
60+	663 (61.7)	1		188 (15.3)	1	
**Education**						
9 year	516 (57.8)	°1.13	(0.94-1.36)	167 (18.5)	*0.76	(0.59-0.97)
10 year	707 (56.4)	°1.12	(0.95-1.33)	293 (23.5)	°0.87	(0.70-1.08)
12+ year	953 (52.4)	1		446 (25.5)	1	

* = significant p < 0.001, ** = significant p < 0.05, ° = not significant.

### Presenteeism with symptoms of a common cold

The proportion of people always reporting to work despite of showing symptoms of a cold was eased from 50.8% in 2008 to 40.9% in 2009. Sex, age group and education do not make a significant difference for this question.

### Hygiene masks

No significant increase was found with regard to the acceptance of hygiene masks from 2008 to 2009. Neither significant difference between women and men was found for this question. Younger age groups showed a lower acceptance compared to the most elderly. The lower educated group did accept hygiene masks more frequently than the most highly educated (Table 2).

**Table 2:** Multivariate analysis of factors associated with propensity for presenteeism and acceptance of hygiene mask (n = 4.012, pooled data week 49–51 2008 and week 48 2009)

	**Always report to work when caught a cold**^**€**^			**Hygiene masks should be applied in public**		
	**No./%**	**Odds ratio**	**Confidence interval 95%**	**No./%**	**Odds ratio**	**Confidence interval 95%**
**Year**						
2009	428 (40.9)	*0.66	(0.53-0.82)	692 (36.4)	°1.17	(0.99-1.39)
2008	501 (50.8)	1		601 (32.6)	1	
**Sex**						
Female	406 (44.8)	°0.75	(0.78-1.22)	694 (35.3)	°1.06	(0.89-1.26)
Male	523 (46.3)	1		599 (33.7)	1	
**Age**						
18-29	137 (38.6)	°0.70	(0.41-1.20)	143 (24.0)	*0.43	(0.32-0.58)
30-44	369 (46.2)	°0.95	(0.58-1.55)	335 (29.7)	*0.59	(0.47-0.73)
45-59	359 (48.9)	°1.05	(0.64-1.72)	371 (36.8)	**0.77	(0.62-0.96)
60+	64 (46.8)	1		444 (43.1)	1	
**Education**						
9 year	160 (49.0)	°1.26	(0.96-1.65)	344 (38.6)	**1.22	(1.00-1.48)
10 year	311 (44.6)	°1.09	(0.88-1.35)	386 (31.3)	°1.01	(0.85-1.20)
12+ year	450 (42.7)	1		534 (30.1)	1	

* = significant p < 0.001, ** = significant p < 0.05, ° = not significant, ^**€**^ = working people only/n = 1937.

## Discussion

Three of the four tested indicators have shown significant changes over the period of the survey. Perceived efficacy of hand washing and preference for coughing into the sleeve have increased significantly, while propensity for presenteeism has decreased. The acceptance of hygiene masks has remained on a stable level. How do the observed changes relate to the results to the institutional hygiene-recommendations in Germany during influenza A H1N1 pandemic? All three observed changes are in accordance with the federal hygiene recommendations. And as there was no official recommendation to wear a hygiene mask, but only an explanation, that there is not enough evidence found yet to recommend or not recommend it, the hold of perception in this issue can be considered consistent with the given information by federal public health institutions, too [10].

By far the biggest increase concerned the perception of coughing into the sleeve as best way to cough. One reason for this could be the low starting value with only a 4.8% acceptance rate and the following high awareness for this as the most novel one of the hygiene recommendations in 2009. Compared with the total acceptance of other recommendations, the 38.3% acceptance of coughing into the sleeve could possibly increase even further with future hygiene promotion activities.

A straight stimulus-response-model of communication cannot be appropriate for the interpretation of the consistence of the institutional hygiene recommendations and the hygiene perception changes found in this study. Almost twelve months of societal discourse about how to avoid influenza infections must be considered as well. We must assume that the general extensive media coverage of influenza and infection control during the influenza A H1N1 pandemic contributed in boosting hygiene perception in Germany. Federal institutions played their role as one contributor of information to be discussed by multiple stakeholders in the communication process, e.g. journalists, editors of web- and social media publications, employers, medical professionals, teachers, to mention but some. Therefore this study rather highlights the results of this societal discourse and provides a reference for future studies in the field.

The results indicate that some sub-groups tend to have a different hygiene perception compared to others. Initially, this can be interesting from a sociological perspective, e.g. results showed that women estimate hand washing as more effective and state more frequently that it is best to cough into the sleeve than men. This corresponds to the higher compliance to prevention in general, which was indicated by former qualitative research [17].

Following these insights it seems beneficial to explore specific informational needs of sub-groups in order to provide customized information. For instance an experimental study found different forms of hand wash promotion to be effective for men and women. While disgust was the most important driving force to make men wash their hands, it did not help to motivate women. It appears ideal then to have different initiatives for women and men [18]. Yet, if it finally comes to risk and crisis communication, a popular recommendation for communicators is that their activities should stay factual and avoid emotionally loaded words [19]. This contradiction poses the question as to whether risk and crisis communication in general and pandemic communication in particular can benefit from heterogeneous and emotional approaches or not. However, the more stratified the communication strategy becomes, the less practical it will to be implemented in the hurry of an acute outbreak. Our results are rather supportive for keeping communication strategies less stratified: As we could not find any interaction of time and demographics, we see all sub-groups’ hygiene perception has benefitted from the public discourse during influenza A H1N1 pandemic. Especially under the time-pressure of an extraordinary outbreak the benefits of stratification can become questionable due to a lack of rapid feasibility.

Public health agencies are in charge of their contributions such as timely, factual and consistent messages, but cannot take responsibility for societal discourse on the whole. Communication strategies that invite the population from the beginning to dialogue and to further spread the word will be less at risk to fail. According to this, the found hygiene perception changes can be understood as a result of all contributions to the hygiene related discourse during influenza A H1N1 pandemic in Germany.

As far as we know, no other pre-post study on hygiene perception for this time-period has been published. The general barrier for this type of study design is that a pandemic outbreak cannot be predicted. Therefore a baseline study is hard to provide and in most cases other study designs have to be found. For instance cross-sectional studies with two waves decided to compare *during* vs. *after* the pandemic peak [20]. Other studies deal with the problem by asking, retrospectively, whether the respondent has changed attitude or behaviour since the beginning of the pandemic [21,22]. Cross-sectional studies with short and frequent intervals, especially in the beginning of an outbreak when everybody forms opinion about the new situation, will probably have a better chance on exploring factors directly associated with hygiene perception and dynamics of changes [23-25]. This study design will be the most demanding one in resources, though. If the research question is mainly about hygiene behaviour, another promising approach is to access existing data which can be used as an indicator for hygiene issues. A study using the incidence of keratoconjunctivitis as an indicator for hand hygiene has revealed remarkable results yet [26].

Hygiene education tends to be considered something to go through as a young child, but which is no longer an issue for adults. This study documents a significant increase in hygiene perception in adults in accordance with the campaign’s hygiene recommendations during influenza A H1N1 pandemic. Such improvements imply that hygiene must not be considered an issue for the education of children alone, but rather for lifelong learning or at least lifelong not forgetting. Public health professionals will have to find a balanced approach of reminding adult people of the measures and benefits of hygiene in their daily life.

The objective of hygiene promotion is not only to improve the standing of hygiene, but also the hygiene behaviour of the population and finally the protection against infections. A correlation between the indicators of the described questionnaire, the resulting hygiene behaviour and the resulting decrease of infections has yet to be proven. If we assume that a higher awareness at least partially leads to improved hygiene behaviour, this raises the question: did hygiene behaviour influence the progression of the pandemic? The general effect of non-pharmaceutical interventions has been described widely [1]. Efficacy of hand hygiene specifically for influenza A H1N1 virus has been reported yet [27]. Future studies should also come up with ideas how to measure the efficacy of these hygiene interventions for a pandemic setting.

### Limitations

A possible bias on our results could be that the general public awareness for the influenza A H1N1 pandemic has possibly led to another level of understanding concerning the questions of this survey. In the German language, the ideas of *Grippe* [flu], *Erkältung* [common cold] and *grippaler Infekt* [acute respiratory infection] are closely related. Therefore a substantial spread in the individual interpretation of symptoms has to be taken into account. During the pandemic, the questions could be more likely interpreted related to the more threatening disease. In this case, the increase of hygiene perception would stem at least partially from the increase of the perceived threat. In comparison, the mild course of pandemic influenza in Germany did not excessively nourish the pandemic threat.

A general problem also plays an important role in this study. To ask questions concerning hygiene may result in people feeling they are being tested by the interviewer. Some respondents might even try to work out the (right) answer that is expected, instead of giving an authentic response. Respondents might presume that some answers are more acceptable to the interviewer than others [28]. We tried to find expressions that did not imply that some answers might appear more correct or acceptable than others. However, the observed changes from this study might be influenced by an increased awareness for hygiene as a social norm during the influenza A H1N1 pandemic.

## Conclusions

The results revealed changes in hygiene perception during influenza A H1N1 pandemic in Germany. The changes we found are in accordance with the recommendations about hand hygiene, coughing into the sleeve and staying home from work while showing symptoms of a cold, as given by the federal campaign *Wir gegen Viren* [Us against viruses] [10]. The results can constitute a practical benchmark for general future research about hygiene perception of adults as well as for tracking hygiene perception changes in context of hygiene promotions. As hygiene perception increased during influenza A H1N1 pandemic a pivotal question is: does the increase in hygiene perception persist after the influenza A H1N1 pandemic has ceased?

## Competing interests

The authors declare that they have no competing interests.

## Authors’ contributions

GM has made substantial contributions to planning and implementing of the interventions, to the design of the survey and to the analysis and interpretation of data. He has been writing the manuscript. WB has made substantial contributions to planning and implementing of the interventions and the indicators for the study. KR has made substantial contributions to planning and implementing of the interventions and the indicators for the study. TW has made substantial contributions to statistical analysis of data. UM has made substantial contributions to the description of method, to the representative sampling and acquisition of data. CB has made substantial contributions to planning and implementing of the interventions and the indicators for the study. All authors revised the manuscript critically and approved the final version.

## Pre-publication history

The pre-publication history for this paper can be accessed here:

http://www.biomedcentral.com/1471-2458/13/959/prepub
